# MAB21L2, a vertebrate member of the Male-abnormal 21 family, modulates BMP signaling and interacts with SMAD1

**DOI:** 10.1186/1471-2121-5-48

**Published:** 2004-12-21

**Authors:** Danila Baldessari, Aurora Badaloni, Renato Longhi, Vincenzo Zappavigna, G Giacomo Consalez

**Affiliations:** 1 Dept. Neuroscience, San Raffaele Scientific Institute, 20132 Milan, Italy; 2 Stem Cell Research Institute, San Raffaele Scientific Institute, 20132 Milan, Italy; 3 Dept. Molecular Biology and Functional Genomics, San Raffaele Scientific Institute, 20132 Milan, Italy; 4 National Research Center, Institute of Chemistry of Molecular Recognition, 20131 Milan, Italy

## Abstract

**Background:**

Through *in vivo *loss-of-function studies, vertebrate members of the Male abnormal 21 (mab-21) gene family have been implicated in gastrulation, neural tube formation and eye morphogenesis. Despite mounting evidence of their considerable importance in development, the biochemical properties and nature of MAB-21 proteins have remained strikingly elusive. In addition, genetic studies conducted in *C. elegans *have established that in double mutants *mab-21 *is epistatic to genes encoding various members of a Transforming Growth Factor beta (TGF-beta) signaling pathway involved in the formation of male-specific sensory organs.

**Results:**

Through a gain-of-function approach, we analyze the interaction of *Mab21l2 *with a TGF-beta signaling pathway in early vertebrate development. We show that the vertebrate *mab-21 *homolog *Mab21l2 *antagonizes the effects of *Bone Morphogenetic Protein 4 *(*BMP4*) overexpression *in vivo*, rescuing the dorsal axis and restoring wild-type distribution of *Chordin *and *Xvent2 *transcripts in *Xenopus *gastrulae. We show that MAB21L2 immunoprecipitates *in vivo *with the BMP4 effector SMAD1, whilst *in vitro *it binds SMAD1 and the SMAD1-SMAD4 complex. Finally, when targeted to an heterologous promoter, MAB21L2 acts as a transcriptional repressor.

**Conclusions:**

Our results provide the first biochemical and cellular foundation for future functional studies of mab-21 genes in normal neural development and its pathological disturbances.

## Background

The *male-abnormal 21 *(*mab-21*) gene was first characterized in *Caenorhabditis elegans *as part of the combinatorial genetic code affecting morphogenesis of the sensory rays, a male-specific sense organ located in the tail and involved in copulation. In that context, early studies [1] identified a hypomorphic *mab-21 *allele as a dominant enhancer of *egl-5*, a loss-of-function mutation affecting the nematode homolog of the *Drosophila *homeotic gene *AbdominalB*. Subsequent studies [2] demonstrated that *mab-21*(+) plays a key role in the formation of the nematode male tail. Homozygous *mab-21 *mutations act cell-autonomously on the identity of a sensory ray, leading to alterations in anteroposterior identities akin to a homeotic transformation. Furthermore, they act non-cell-autonomously on the binary switch between hypodermal and neuroblast specification. Finally, hypomorphic *mab-21 *mutants exhibit pleiotropic changes affecting movement, body shape and fecundity, suggesting that *mab-21*(+) also plays key developmental roles outside the male tail region.

The *mab-21 *gene encodes a 41 kD basic protein with no significant homologies to any other published proteins. Homologs of the *mab-21 *gene have been identified in many other species, from *Drosophila *to humans. The first vertebrate counterparts of *mab-21 *were isolated in amniote genomes: a human homolog, *MAB21L1*, was cloned during a systematic search for transcripts carrying trinucleotide repeats [3] and potentially involved in the pathogenesis of neuropsychiatric disorders. Interestingly, recent physical mapping data have located *Mab21l1 *within a chromosomal deletion discovered in a patient with autism and language deficit secondary to an auditory processing abnormality [4].

Our group, in parallel with others, identified two murine *mab-21 *genes (*Mab21l1*, *Mab21l2*) [5-8] and the human *MAB21L2 *gene, and showed that they encode nuclear proteins [6] that are 94% identical, 98% homologous to each other. In the mouse, *Mab21l1 *and *Mab21l2 *are expressed in largely overlapping territories [5-7], suggesting the possibility of a high degree of functional redundancy. More recently, only one vertebrate member of the mab-21 family was isolated in *Xenopus laevis *(*Xmab21l2*) [9], (and our unpublished data: GenBank entry AF040992) and *Danio rerio *[10,11] (*Zmab21l2*). *Mab21l2 *is an early marker of the tectum as well as primitive eye field, optic cup and retina in anamniotes, in agreement with observations previously made in mouse embryos.

To address the *in vivo *roles of mab-21 genes in vertebrates, loss-of-function studies have been carried out by different groups in *Xenopus *and mouse embryos. Lau et al. [9] interfered with the functions of *Xmab21l2 *in embryogenesis by applying antisense DNA and double-stranded RNAi techniques. As a result, they reported a high frequency of embryos arrested at late gastrula/early neurula, as well as a significant incidence of neural tube closure defects in tadpoles. Likewise, Wong and Chow [12] cultured mouse embryos in the presence of antisense oligos specific for *Mab21l1 *and *Mab21l2*, and reported that both treatments caused a sharp increase in the incidence of defective axial turning, incomplete notochord formation, and neural tube closure defects. However, *Mab21l2*-specific antisense oligos were more potent and more effective than *Mab21l1*-specific ones, suggesting that *Mab21l2 *may play a more irreplaceable role in early development than *Mab21l1*.

As said, mab-21 genes mark early stages of eye development in various species. Likely due to redundancy in the vertebrate mab-21 gene family a knock-out mouse carrying a null mutation of *Mab21l1 *featured an isolated, cell-autonomous defect in lens placode development, providing a novel and valuable model for the study of various eye defects in humans [13], but no overt retinal abnormalities. Conversely, *Mab21l2 *has been found to participate crucially in zebrafish retina formation acting as a downstream effector of *Rx2 *[14].

Some of these important advances point to a critical role for the mab-21 gene family as a whole in early embryogenesis on one hand, and in various aspects of neural development on the other. However, our knowledge on the molecular mechanisms through which mab-21 genes operate remains inadequate at best. Interestingly, by genetic analysis of loss-of-function mutations in *C. elegans*, Morita *et al*. have proposed *mab-21 *as a downstream target of a TGF-beta superfamily signaling pathway involved in sensory ray identity, the Small/Male-tail-abnormal pathway. This pathway is initiated by the secreted ligand CET-1 [15], also known as DBL-1 [16], and regulates body growth and male tail development. The CET-1/DBL-1 ligand transmits its signal through two receptor serine threonine kinases, DAF-4 and SMA-6, which in turn regulate the activity of the nuclear transducers SMA-2, SMA-3, and SMA-4. Hypomorphic mutations of *mab-21 *are epistatic to cet-1 pathway mutations affecting ray pattern formation (*cet-1*, *sma-2*, *sma-3*, *sma-4*), while they do not affect body size in double mutants [15]. Reportedly, this interaction did not reflect regulation of *mab-21 *gene transcription by the sma signaling pathway.

The above genetic studies prove the biological relevance of the *mab-21 *– TGF-beta interaction, but stop short of addressing several key questions. What is the molecular counterpart of the genetic interaction described in *C. elegans *between *mab-21 *and the sma pathway? Is this interaction conserved in vertebrate development, and what TGF-beta superfamily molecules does it involve? Does it play a relevant role in the establishment of vertebrate dorsoventral (DV) polarity? If so, at what level do MAB-21 and TGF-beta pathways converge into a single regulatory cascade? In the present study, through a gain-of-function strategy, we analyze the molecular interplay between MAB21L2, a vertebrate homolog of *C. elegans *MAB-21, and TGF-beta superfamily signaling molecules. In particular, we focus on the signaling pathway initiated by BMP4, a ligand structurally related to CET-1/DBL-1, that regulates DV axis formation and numerous key aspects of neural development in *chordata*.

## Results

### *Xmab21l2 *expression in development

**Figure 1:**
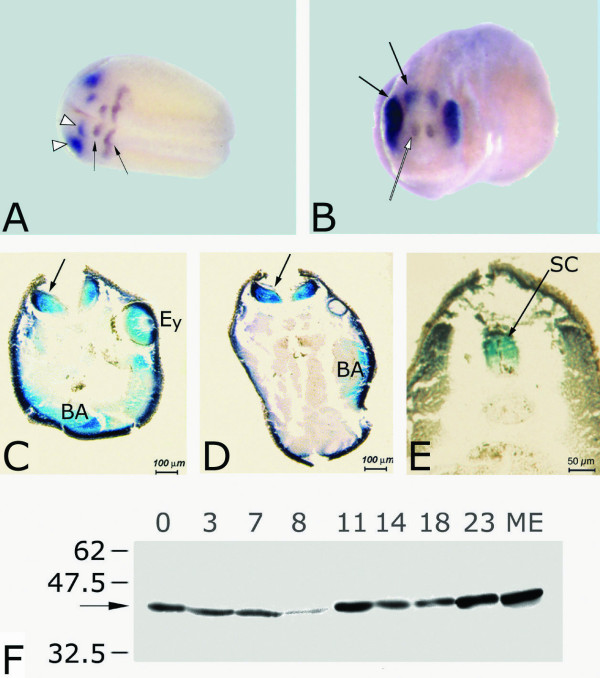
**Expression of Mab21l2 gene and protein in *Xenopus laevis *development. **A) Double whole mount in situ hybridization for *Xmab21l2 *(blue) and *Xkrox20 *(brown). At the end of neurulation (dorsal view of a st. 19 embryo is shown) *Xmab21l2 *expression (arrows) is restricted to the eye primordium (bottom arrowhead) and midbrain (top arrowhead). *Xkrox20 *labels the third and fifth rhombomeres (arrows). B) Frontal view of a stage 24 embryo. *Xmab21l2 *expression (blue, arrows) is restricted to the eye (left, solid arrow) and midbrain (right, solid arrow), clearly posterior to the forebrain marker *Emx2 *expression domain (brown, empty arrow). C–E: Sections obtained from late tadpole embryos were wholemount-hybridized with *Xmab21l2*. Three transverse sections, from anterior to posterior, show *Xmab21l2 *expression in the retina and lens (Ey), in the branchial arches (BA), midbrain (arrow in C), hindbrain (arrow in D), and in the dorsal neural tube, (SC in E). F: Western blot analysis of MAB21l2 protein expression in *Xenopus *oocytes (stage 0) and *Xenopus *embryos (stages 3–23). ME: day 12 mouse embryo (positive control). Protein concentrations were quantitated through a Bradford assay and loaded on gel in equal amounts. The experiment shows maternal expression of MAB21l2 (41 kD, arrow), the levels of which decrease around blastula stage to resume zygotically thereafter. In particular, stage 11 gastrulae are positive for the MAB21l2 protein.

It is well established that the BMP4 pathway is essential for proper formation of the dorsoventral axis; overexpression of *BMP4 *in *Xenopus *embryos can lead to the formation ventralized embryos, i.e. embryos devoid of axial structures, whereas BMP4 depletion can lead to dorsalized embryos, i.e. embryos exhibiting an expansion of dorsal mesoderm [17-20]. At tailbud stages, the expression of *Xmab21l2 *is restricted to the primitive eye field, optic recess, optic cup and retina (excluding the choroid fissure), to the tectum and dorsal neural tube, and to branchial arches (Figure 1A–E). The early-onset and sustained expression of the MAB21L2 protein in oocyte-, blastula, gastrula, tailbud and tadpole lysates suggests that *Mab21l2 *is expressed both maternally and zygotically, during and after gastrulation (Figure 1F).

### Mab21l2 coexpression rescues the dorsal axis in BMP4-injected Xenopus embryos

To determine whether *Xmab21l2 *and *BMP4 *have a synergistic or antagonistic interaction *in vivo*, we took a gain of function approach utilizing *Xenopus laevis *embryos. Preliminary RT-PCR experiments conducted on gastrula lysates injected at the one-cell stage with *BMP4 *mRNA had shown that, as previously reported by others in studies conducted in the nematode, *Xenopus Mab21l2 *expression is not controlled by BMP4 signaling (not shown).

**Figure 2:**
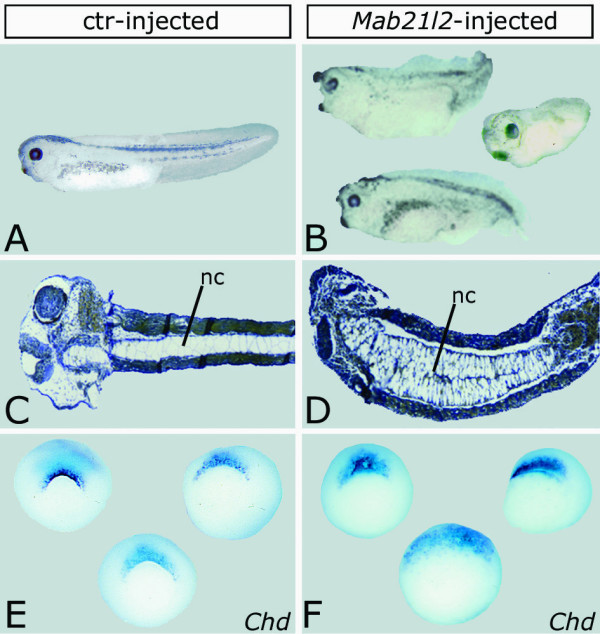
**Isolated overexpression of *Xmab21l2 *produces partially dorsalized embryos. **A, C, E: control-injected embryos; B, D, F: *Xmab21l2*-injected embryos. A–D: tailbud-tadpole stages; E, F: gastrula stage. In B: external appearance of *Xmab21l2*-injected embryos. D: horizontal section of an *Xmab21l2*-injected section stained with orange-G/aniline blue. Note enlarged notocord (nc). F: expanded and enhanced *Chordin *expression in *Xmab21l2*-injected gastrulae (blue signal).

Prior to addressing the nature of the interaction between *Xmab21l2 *and BMP signaling molecules, we analyzed the effects of isolated *Xmab21l2 *overexpression. Embryos were injected at the one-cell stage with 800 pg *in vitro*-transcribed *Xmab21l2 *alone. We analyzed the phenotype of injected tailbud embryos as well as the expression of dorsal markers at gastrulation. A significant percentage of injected embryos ***(48%, N = 200) ***featured various degrees of dorsalization/anteriorization (DAI 6–8), with a well formed, sometimes enlarged head, a broad cement gland, a short and/or bent axis (Figure 2B). We prepared embryos for histological examination by sectioning them along the longitudinal plane, both sagittally and horizontally. Histological analysis of horizontal sections revealed many tadpoles featuring an enlarged notochord (nc, fig. 2D), suggestive of a possible defect in convergent extension. Finally, wholemount *in situ *hybridization experiments conducted on gastrulae with markers of DV polarity revealed an increased percentage of embryos (50%, N = 22) with expanded and/or enhanced *Chordin *gene expression (blue signal, fig. 2F). Perturbation of *Xvent2 *gene expression was sporadic, with 5 out of 21 *Xmab21l2*-injected embryos featuring a reduced *Xvent2 *expression domain (not shown). Our results are in sufficient agreement with those previously obtained by others [9], confirming that *Xmab21l2 *overexpression produces neither signs of complete dorsalization, such as Janus twins or radial eyes [21], nor axis duplication.

**Figure 3:**
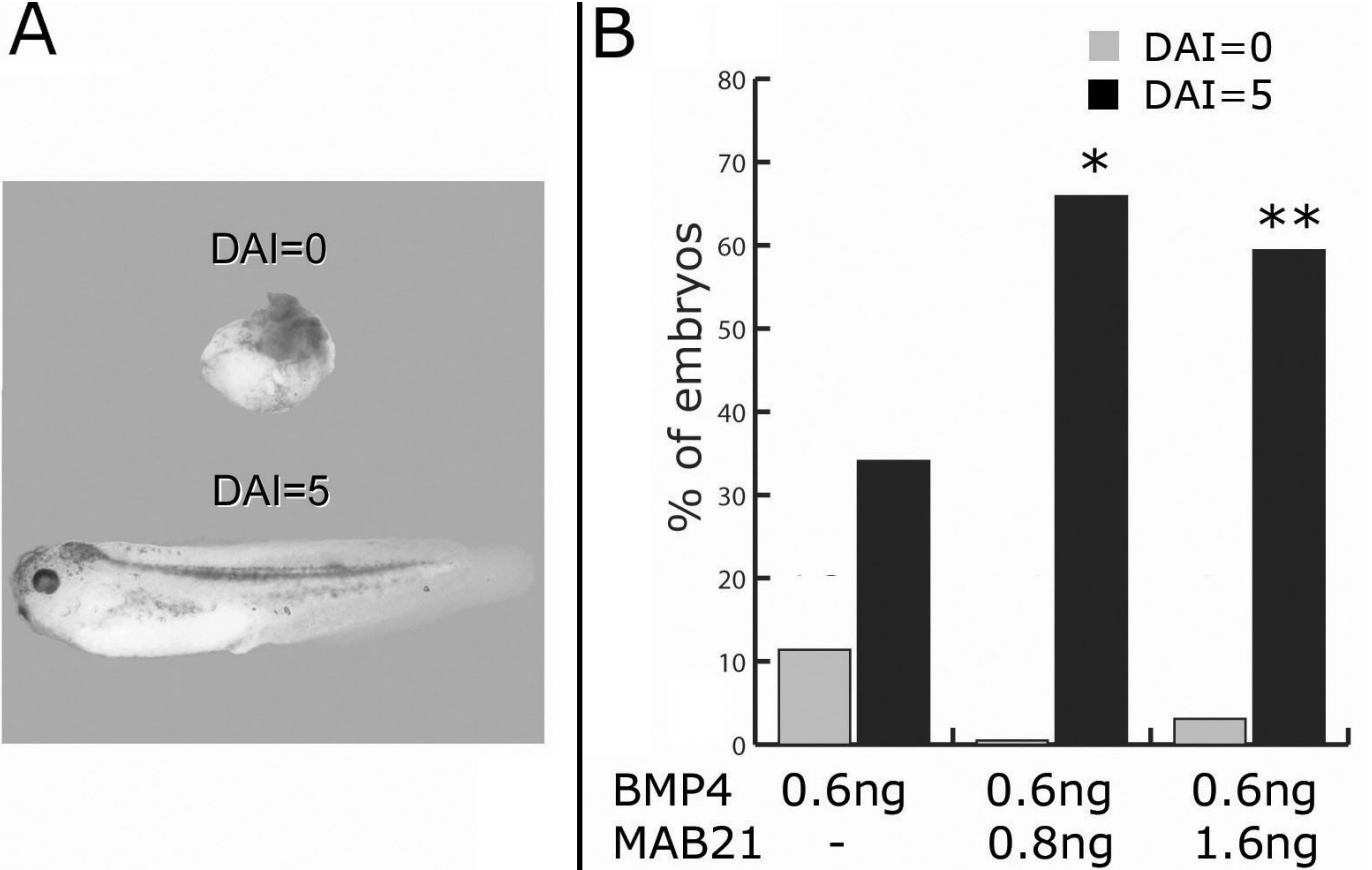
***Xmab21l2 *rescues dorsal structures in *BMP4 *injected embryos. **(A) As previously described, injection of 0.6 ng of *BMP4 *mRNA gave rise to ventralized embryos. The most severely ventralized phenotype of BMP4 injection, called *bauchstück*, was scored with a dorso-anterior index (DAI) of 0 (top), whereas normally dorsalized embryos were assigned a DAI of 5 (bottom). (B) Embryos were injected with 0.6 ng BMP4 (n = 111), with 0.6 ng BMP4 plus 0.8 ng Mab21l2 (n = 83), or with 0.6 ng BMP4 plus 1.6 ng Mab21l2 (n = 101). In each group, we scored the percentage of complete ventralization (DAI = 0, grey bars) and normal dorsal axis formation (DAI = 5, black bars) in embryos that completed development. Intermediate classes are omitted in the plot. Coinjection of embryos with *Mab21l2 *significantly increased the percentage of correctly dosalized embryos (see text for details). Statistical analysis was conducted using the Chi square algorithm (1 *df*). *: *p *= 0.0005; **: *p *= 0.0087.

To determine whether *Xmab21l2 *can compensate the effects of *BMP4 *overexpression, *Xenopus *embryos were injected at the one-cell stage with *in vitro*-transcribed *BMP4 *mRNA alone (0.6 ng, *n *= 111), or coinjected with *Xmab21l2 *mRNA (0.8 ng, *n *= 83; 1.6 ng, *n *= 101). We scored the formation of dorsal structures in tadpole stage embryos by using the dorsoanterior index [21]. A complete loss of dorsal structures (DAI = 0) (top in Figure 3A) was observed in 11.4% of the embryos after *BMP4 *overexpression (0.6 ng) (Figure 3B). Coinjection with 0.8 or 1.6 ng *Xmab21l2 *drastically reduced this class (0% and 3.1%, respectively). Likewise, whilst complete dorsal structures (DAI = 5) (Figure 3A, bottom) were observed in only 34.1% of *BMP4*-injected embryos, coinjection with 0.8 or 1.6 ng *Xmab21l2 *increased this class significantly (65.9% and 59.4%, respectively), suggesting that *Xmab21l2 *antagonizes *BMP4 *in its ability to ventralize embryonic mesoderm *in vivo *(see Figure 3B for details).

**Figure 4:**
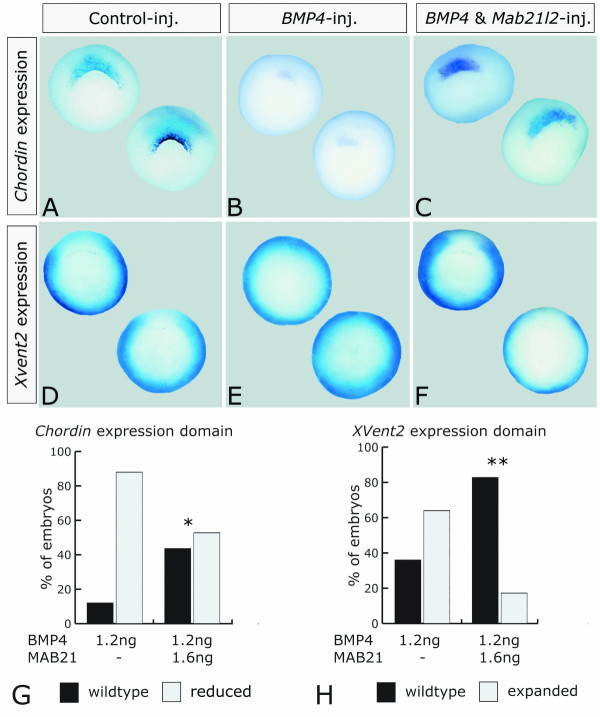
***Xmab21l2 *restores the normal expression of *Xvent2 *and *Chordin *in *BMP4*-injected embryos. ***Chordin *(A–C) and *Xvent2 *(D–E) expression were analyzed by whole mount *in situ *hybridization. Embryos are shown in a vegetal view, dorsal to the top, ventral to the bottom. (A, D) Wild type expression of *Chordin *(A) and *Xvent2 *(D) in stage 10.5 gastrulae; (B, E) embryos injected with 1.2 ng of *BMP4 *mRNA alone showed reduced *Chordin *expression (B) (22/25) and expanded *Xvent2 *expression (E) (16/25); (C, F) embryos co-injected with 1.2 ng of *BMP4 *and 1.6 ng *Xmab21l2 *mRNA showed a rescue of wild type *Chordin *(C) (17/36) and *Xvent2 *expression (F) (24/29). (G) Percentage of embryos exhibiting wild type (black bars) or reduced (grey bars) *Chordin *gene expression in embryos injected with 1.2 ng BMP4 alone, and in embryos coinjected with 1.6 ng *Mab21l2*. Coinjection of *Mab21l2 *significantly increased the number of embryos exhibiting a wild type *Chordin *expression pattern; *: *p *= 0.004. (H) Percentage of embryos exhibiting wild type (black bars) or expanded (grey bars) *Xvent2 *gene expression in embryos injected with 1.2 ng BMP4 and in embryos coinjected with 1.6 ng *Mab21l2*. Again, *Mab21l2 *significantly increased the number of embryos exhibiting a wild type *Xvent2 *expression pattern; **: *p *= 0.00044. Statistical analysis was conducted using the Chi square algorithm (1 *df*).

Next, by wholemount *in situ *hybridization, we analyzed the expression of molecular markers of dorsal (*chordin*, Figure 4A–C) and ventral (*Xvent2*, Figure 4D–F) mesoderm in wildtype embryos, as well as embryos injected with *BMP4 *alone or coinjected with *Xmab21l2*. Whilst the expression domain of *chd *was sharply reduced in 88% of *BMP4*-injected embryos, 47% of the embryos co-injected with *Xmab21l2 *showed wild type *chd *expression (Figure 4B,C,G). Likewise, 64% of the *BMP4*-injected embryos showed a dorsal expansion of *Xvent2 *expression, that was rescued in 83% of the embryos co-injected with *Xmab21l2 *(Figure 4E,F,H).

### MAB21L2 interacts with SMAD1 *in vitro *and *in vivo*

**Figure 5:**
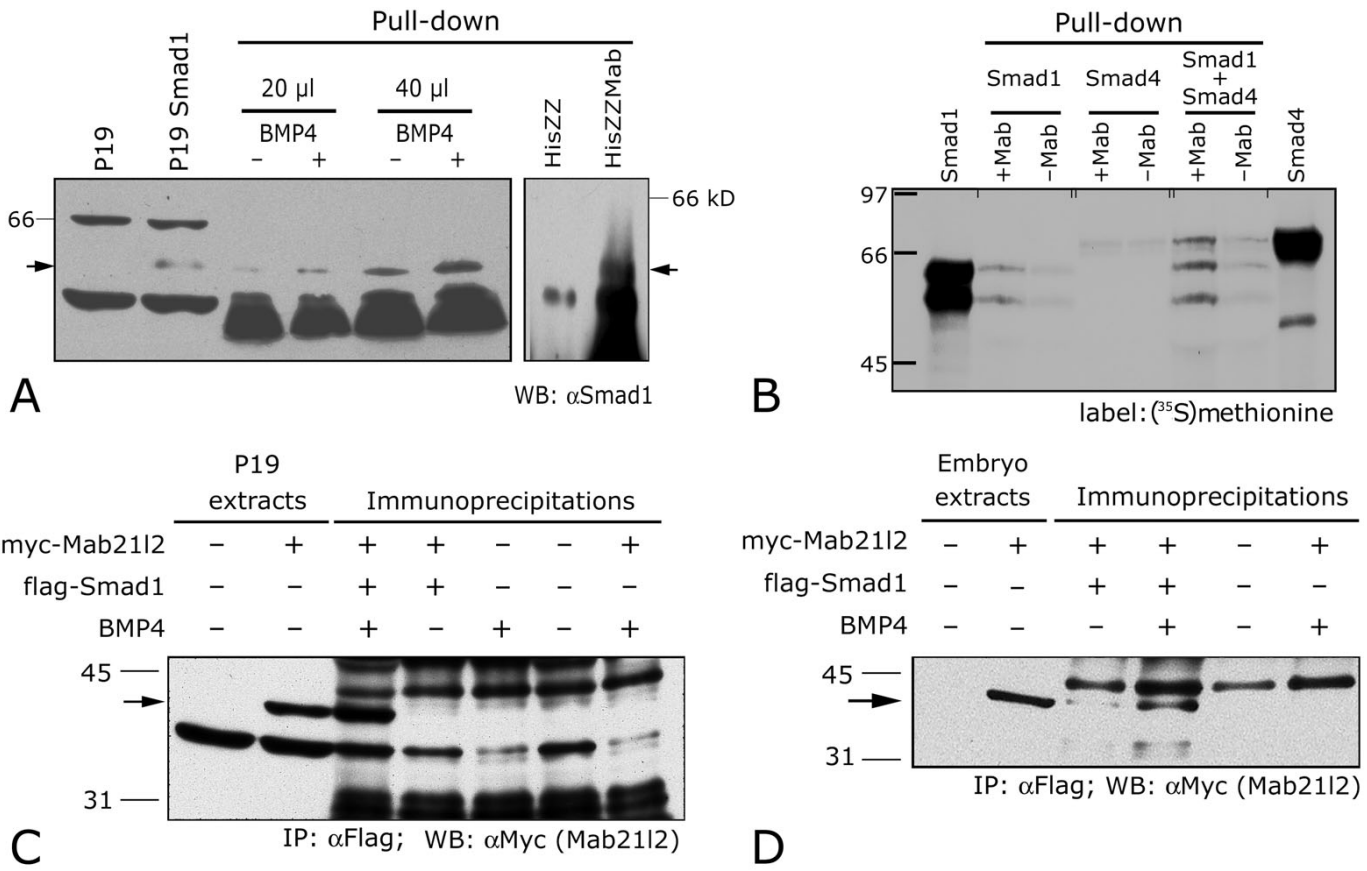
**Mab21l2 interacts with Smad1 *in vitro *and *in vivo. *(A) **Lanes 1, 2: immunoblotting of total lysates from P19 cells, mock- and *Smad1*-transfected, respectively. P19 cells do not express detectable levels of endogenous SMAD1. Lanes 3–8: Affinity chromatography (pull-down) experiment. High-stringency eluates from untransfected P19 were separated by SDS-PAGE and transfered onto nitrocellulose filters. The blots were immunolabeled using an anti-SMAD1 antibody. Lanes 3–6: the *in vitro *interaction is not strictly dependent on activation of BMP signaling: the pull-down experiment was performed with 20 μl (3, 4) and 40 μl of P19 cell lysates (5, 6). Lysates came from P19 cells treated (4, 6) or untreated (3, 5) with BMP4. Lanes 7, 8: an His-ZZ-MAB21L2 protein (HisMab) synthesized in *E. coli *(lane 8) was coupled to a sepharose-Ig resin and incubated with cell lysates of P19 cells treated with BMP4. As a negative control, an *in-vitro *synthesized His-ZZ incubated with the same P19 lysates fails to pull down a 53 kD band. Arrows: SMAD1 (53 kD). **(B) **direct interaction between SMAD1 and MAB21L2; no direct interaction between SMAD4 and MAB21L2. An His-ZZ-MAB21L2 protein (+Mab) synthesized in *E. coli *(lane 2) was coupled to a sepharose-Ig resin and incubated with *in vitro*-translated SMAD1 (lanes 2), SMAD4 (lanes 4), and SMAD1 + SMAD4 (lanes 6). Negative controls were represented by His-ZZ-coupled resins (-Mab) incubated with the same *in vitro*-synthesized proteins (lanes 3, 5, 7). Lanes 1 and 8 contain *in vitro*-synthesized SMAD1 and SMAD4, respectively. **(C) **BMP4-dependent *in vivo *co-immunoprecipitation of flag-SMAD1 and myc-MAB21L2 in P19 cells. Cells were mock-transfected, transfected with flag-SMAD1 and/or with myc-MAB21L2, as indicated, and either treated with BMP4 or left untreated. In lanes 3–7, cell lysates were immunoprecipitated with a monoclonal anti-flag antibody. Cell lysates in lanes 1, 2 and immunoprecipitates in lanes 3–7 were gel fractionated and transfered to nitrocellulose filters. Blots were immunolabeled with an anti myc antibody. Arrow (42 kD) points to a band in lanes 2, 3) corresponding to myc-MAB21L2. **(D) ***in vivo *co-immunoprecipitation of flag-SMAD1 and myc-MAB21L2 in stage 11 *Xenopus *embryos, facilitated by BMP4 overexpression. Embryos were water-injected, injected with *flag-Smad1 *and/or with *myc-Mab21l2 *RNA, as indicated, and coinjected with *BMP4 *where indicated. In lanes 3–7, cell lysates were immunoprecipitated with a monoclonal anti-flag antibody. Embryo lysates in lanes 1, 2 and immunoprecipitates in lanes 3–6 were gel fractionated and transfered to nitrocellulase filters. Blots were immunolabeled with an anti myc antibody. Arrow (42 kD) points to a band in lanes 2–4) corresponding to myc-MAB21L2.

The antagonistic interaction between *Mab21l2 *and *BMP4 *may be either interpreted as evidence of a molecular interaction between MAB21L2 and some BMP signaling transducer, or alternatively suggest that MAB21L2 and some component of the BMP signaling pathway compete for a common cofactor. It is well established that the DNA-binding protein SMAD1, once phosphorylated at the plasma membrane by the BMP receptor heterotetramer, assembles with the receptor-independent protein SMAD4, enters the nucleus, establishes interactions with various cofactors, and acts by regulating the transcription of a host of target genes [reviewed in [22]]. Because vertebrate MAB-21 proteins are mainly or exclusively distributed in the nucleus [6], we investigated the possibility of a molecular interaction between MAB21L2 and SMAD proteins. In order to obtain *in vitro *evidence of this interaction, P19 cells were either treated with BMP4 or left untreated, and subsequently lysed. P19 cell extracts were then incubated with a resin-bound His-ZZ-tagged form of MAB21L2, or with a control His-ZZ-coupled resin. Eluates were analyzed by immunoblotting with a polyclonal anti-SMAD1 antibody. The experiment revealed a clear interaction between synthetic His-ZZ-MAB21L2 and endogenous SMAD1 that was absent when P19 lysates were incubated with the control resin (Figure 5A). Although BMP4 treatment seemed to enhance it considerably, the interaction was not entirely BMP4-dependent, suggesting that conformational changes secondary to receptor-mediated phosphorylation may not be strictly required for SMAD1 to interact with MAB21L2.

These results prompted more questions, such as whether the interaction between MAB21L2 and SMAD1 is direct and whether it is dependent on prior formation of a SMAD1-SMAD4 complex. Once again, we resorted to affinity chromatography, incubating *in vitro*-translated, ^35^S-Met-labeled SMAD1 and SMAD4 with a resin containing His-ZZ-MAB21L2. This experiment suggested that the interaction with SMAD1 is direct, and that MAB21L2 does not assemble with SMAD4 directly but only through SMAD1, indicating that the formation of a SMAD4-MAB21L2 complex is mediated by SMAD1 (Figure 5B). Indirectly, this experiment also showed that MAB21L2 does not obviously compete with SMAD4 to assemble with SMAD1.

Finally, we investigated whether the *in vitro *interaction observed between MAB21L2 and SMAD1 could be replicated *in vivo*. P19 cells co-transfected with myc-MAB21L2 and flag-SMAD1 were either treated with BMP4 or left untreated. In parallel, *Xenopus laevis *embryos were injected at the four-cell stage into the animal pole of each blastomere with *myc-Mab21l2 *and flag-*Smad1 *mRNAs, and coinjected with either *BMP4 *mRNA or H_2_O. The embryos were grown until mid-gastrula stage (st. 10.5/11). Both cell- and embryo lysates were subjected to immunoprecipitation, using an anti flag monoclonal antibody, whereas an anti-myc antibody was utilized for immunodetection in Western blotting. Experiments conducted in transfected P19 cells showed that SMAD1 coprecipitates with MAB21L2, and that the interaction is enhanced by activation of BMP signaling, which is required for nuclear localization of receptor-dependent SMADs (Figure 5C). Likewise, MAB21L2-SMAD1 co-precipitation took place especially, albeit not exclusively, in BMP4-coinjected embryos (Figure 5D). Interaction of the two proteins in the absence of exogenous BMP4 can likely be explained by the abundance of endogenous BMP2/4 ligands in stage 11 gastrulae.

### When targeted to an heterologous promoter, MAB21L2 acts as a strong transcriptional repressor

**Figure 6:**
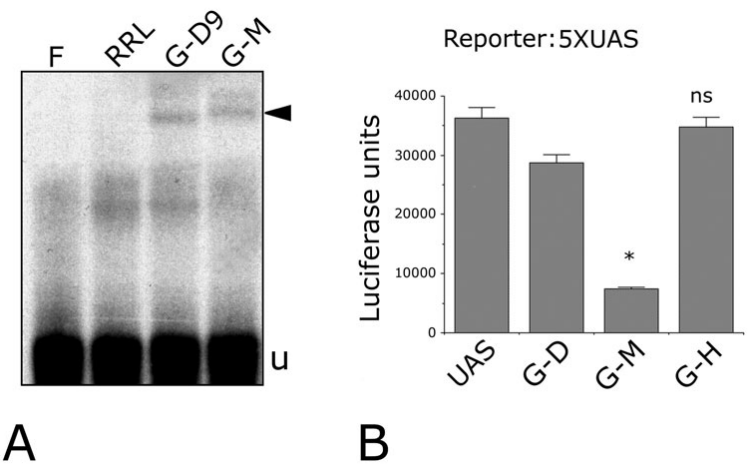
**When targeted to a heterologous promoter, *Mab21l2 *behaves as a strong transcriptional repressor. (A) **An *in vitro*-translated chimeric protein containing the GAL4 DNA binding domain fused to the N-terminus of MAB21L2 was used in an electrophoretic mobility shift assay. An end-labeled ds-DNA containing the GAL4 nucleotide binding site (5XUAS) was incubated in native conditions with *in vitro *translated GAL4-MAB21L2 (G-M) and separated electrophoretically by nondenaturing PAGE. As negative controls, we used free oligonucleotides (F) and rabbit reticulocyte lysates (RRL). As a positive control we used a previously tested GAL4 fusion protein, GAL4-D9NT (G-D9) [40]. The arrow indicates a bandshift specific for GAL4-MAB21L2 and absent in negative controls. u: unbound. **(B) **5XUAS-luc transcription is clearly downregulated in cells transfected with GAL4-MAB21L2 (G-M) vs. untransfected COS7 cells or same cells transfected with GAL4 DBD, G-D) alone (*: *p *= 0.0019). No downregulation of 5XUAS-luc activity was observed in COS7 cells transfected with a fusion of GAL4 and the N-terminal domain of the HOXB3 protein, which possesses no transcriptional activation or repression activity [24] (ns: not significant). Statistical analysis was conducted using the T test method (two tails).

Because *in vivo *MAB21L2 acts as a BMP4 antagonist, and because BMP4 signaling is transduced in the nucleus by SMAD transactivators, we asked whether MAB21L2 has any intrinsic repressor functions. To address this point, we generated fusion transcripts adding the GAL4 DNA binding domain (residues 1–147) [23], either to the N- or C-terminus of MAB21L2. For as yet unexplained reasons, the latter fusion protein proved extremely unstable and was discarded. Conversely, the N-terminal fusion protein is stable and was shown to bind DNA by gel-retardation assay (Figure 6A). Thus, we were able to assess its activity in cotransfection assays, using as a reporter system the GAL4 target promoter (5XUAS) fused to luciferase. Luciferase activity in *Mab21l2*-cotransfected COS7 cells was compared to the baseline reporter activity scored in cells transfected with the GAL4 DBD alone (G-D) or with a transcriptionally inactive GAL4 DBD fusion protein, (GAL4HOXB3NT, G-N) [24]. Reporter activity was clearly downregulated in *Gal4-Mab21l2*-transfected cells only, suggesting that MAB21L2 may function as a transcriptional repressor or co-repressor (Figure 6B). The same luciferase assay was performed in P19 cells, co-transfected with plasmid DNA, G-D, and G-M, revealing a 12-fold downregulation of 5XUAS-luc in G-M-transfected cells (not shown). This confirmed the evidence of a strong repressor activity of MAB21l2 when targeted to DNA.

## Discussion

Mab-21 genes have been isolated in several species and have been shown, through genetic and epigenetic loss-of-function experiments, to play key roles in various developmental processes, ranging from gastrulation and neural tube closure to eye and lens formation [2,9,10,12,13]. However, no biochemical data have been available to identify the regulatory cascade(s) in which these elusive factors are involved. While epistatic analysis has proposed that *C. elegans mab-21 *undergoes inhibitory regulation by TGF-beta superfamily signals [15], the molecular nature of this regulation remained obscure. Likewise, no information has been published as to the conservation and relevance of the sma/mab-21 interaction in vertebrate development.

Our results, obtained in an anamniotic model system, the *Xenopus *embryo, and in murine embryonic carcinoma cells, indicate that MAB21L2 interacts functionally with SMAD1, a nuclear transducer of BMP2/4/7 signaling. Overexpression of *Mab21l2 *complements the effects of *BMP4 *overexpression, in keeping with the epistatic interactions observed in nematodes [15]. Thus, genetic interactions first described in *pseudocoelomates *appear to be strongly conserved in evolution.

As the nuclear localization of MAB21L2 suggested, our pull-down and immunoprecipitation results indicate that MAB21L2 is a new interactor of the receptor-activated transducer SMAD1. Whilst the interaction between MAB21L2 and SMAD1 appears to be direct, our results do not seem to favor a direct contact between MAB21L2 and SMAD4.

The interaction of MAB21L2, a transcriptional repressor, with nuclear transducers of BMP signaling offers a possible explanation for the results of loss-of-function experiments conducted by other investigators in *Xenopus laevis *and mouse embryos. The increasing levels of MAB21L2 protein observed starting at midblastula transition may be required to compensate mesoderm-ventralizing signals triggered by BMP2/4. In the absence of MAB21L2 genes, completion of gastrulation may be hampered, resulting in various abnormalities of dorsoventral polarity [9,12]. Results obtained by others have demonstrated that SMAD complexes can be turned into negative regulators of transcription through a physical interaction with transcriptional repressors [25]. Our results indicate that MAB21L2 possesses a considerable transcriptional repressor activity. However, the antagonistic interactions between MAB21L2 and BMP signaling may not necessarily depend on the formation of transcriptional regulation complexes. Further experiments will be required to characterize the mechanism(s) of transcriptional regulation mediated by MAB21L2. Importantly, the identification of *Mab21l2 *downstream genes will make it possible to investigate *mab-21 *– *BMP4 *interactions from the opposite standpoint, i.e. by looking at the modulatory effect of BMP signaling on the expression of *Mab21l2 *targets.

Although our results are more directly relevant to understanding the functions of *Mab21l2 *in early embryogenesis, the gene is clearly expressed at high levels and in a tightly localized fashion in neurulation and morphogenesis as well, likely contributing to neural tube and eye formation. Indeed, because of redundancy in the mab-21 gene family, many of the early effects of mab-21 gene mutation may be masked, uncovering these genes' roles in later stages of development and postnatal life.

In late developmental processes, well beyond the end of gastrulation, MAB-21 proteins may prolong their dynamic interaction with BMP signaling transducers. As mentioned, a cell-autonomous requirement for *Mab21l1 *in lens formation has been recently documented in knock-out mice [13] that develop otherwise normally in the nervous system. In the same mice, genetic redundancy has hampered the analysis of mab-21 gene function in retinal development. However, antisense studies have shown that inhibition of *mab-21 *leads to disruption of the retinal anlage, where the gene is strongly expressed in a territory overlapping with the *Bmp4 *and *Xvent2 *expression domain. Recent studies have shown that BMP signaling plays a relevant role in guiding morphogenesis along the dorsoventral axis of the chick [26] and *Xenopus *[27] retina. In this context, it would be interesting to determine if mab-21 genes act by balancing BMP signals in the establishment of retinal polarity.

*Mab21l1 *and *Mab21l2 *are prominently expressed in the embryonic midbrain, both dorsally and ventrally, and in prosomere 1 [5,6]. Roles exerted by BMP signaling molecules in these territories are starting to emerge, and appear relevant to the induction and patterning of dorsal mesencephalic structures (roof plate) [28], and, more ventrally, to the differentiation of dopaminergic precursors [29].

Throughout the hindbrain and spinal cord, *Mab21l2 *is expressed mostly in dorsal territories. In cerebellar development, it is expressed in the cerebellar plate and, later on, in granule cell neurons [5]. BMP signaling molecules are coexpressed with *Mab21l2 *at several of those sites, and their developmental roles have been at least partially addressed: as an example, granule cell specification can be induced by BMP7 overexpression in the dorsal and ventral rhombencephalon [30]. BMP7 is homologous to BMP2 and BMP4, and its signal can be transduced by SMAD1 [31]. Likewise, in the spinal cord *Mab21l2 *is expressed in a dorsal territory, where BMP2/4 signaling plays a key role in the specification of neuronal identities alongside the dorsoventral axis of the neural tube [32-34].

Finally, in postmigratory neural crest, the interplay between BMP2/4 and *Mash1 *maintains competence for neuronal differentiation [35]. In neural crest derivatives, including the branchial arches, *Mab21l2 *may thus participate in specifying neuronal and non-neuronal cell fates by interacting with BMP signals.

## Conclusions

Our results provide a biochemical and molecular foundation for future studies of *mab-21 *gene function in vertebrate systems, demonstrating that MAB21L2 interacts functionally with the BMP4 signalling pathway and physically with its best characterized nuclear transducer, SMAD1. Furthermore, we show that MAB21l2 can act as a powerful transcriptional repressor when targeted to an heterologous promoter. Further work is clearly required to determine if MAB21L2 binds DNA, what its binding specificity is, or if it only exerts its effect in a DNA-binding-independent fashion. More broadly, additional studies are required to clarify the role of mab-21 genes in the context of BMP signaling, and their likely function as a molecular switch linking different regulatory pathways in development and disease.

## Methods

### Image acquisition and processing

Images were obtained either by traditional photography (slides 1–3) or by electronic scanning of autoradiography films. All images were processed through the Adobe Photoshop 7.0 software or through the Adobe Illustrator 10 software.

### Isolation of Xmab21 cDNA and constructs

The *Xenopus laevis *homolog of mouse *Mab21L2 *was isolated by screening a *Xenopus *stage 28–30 lambdaZapII library (Courtesy of Richard Harland) using the murine homolog as a probe. The 2.8 Kb clone isolated contained the full length *Xmab21l2 *cDNA (GenBank AF040992) as well as 5' and 3' regions. The coding region of *Xmab21 *was excised using NotI and BalI (1.3 Kb) and subcloned into pBluescript for whole mount in situ hybridization, or BamHI-StuI (1.1 Kb) and subcloned into pT7TS for overexpression in embryos and into pCDNA3 for cell transfection.

### Embryos

All animal experimentation was conducted according to the stipulations of the Institutional Animal Care and Use Committee, San Raffaele Scientific Institute. In order to obtain embryos, *Xenopus *females were primed 1000 U of human chorionic gonadotropin (Profasi HP 5000, Serono) the night before collection. Ovulated eggs were fertilized with testis homogenates and allowed to develop in 0.1X MMR (1X MMR is 0.1 M NaCl, 2.0 mM KCl, 1.0 mM MgCl2, 2.0 mM CaCl2, 5 mM HEPES, pH7.4). Jelly coats were removed in 3.2 mM DTT, 0.2 M Tris pH 8.8. Embryos were staged according to Nieuwkoop and Faber [36] and fixed in MEMFA [37] for in situ hybridization.

### Histology

For histological examination, wt or injected and stained embryos were fixed in MEMFA, embedded in wax, cut into 10 μm sections, dried onto slides, dewaxed with xylene and dehydrated with alcohol. Sections were rehydrated and stained with an orange G solution (2 g orange G, 8 ml glacial acetic acid, 100 ml water) and subsequently with an orange G-aniline blue solution (2 g orange G, 0.5 g aniline blue, 8 ml glacial acetic acid, 100 ml water), and dehydrated [36,38].

### Whole-mount in situ hybridization

Whole-mount in situ hybridization was performed on staged embryos as described by [37]. The antisense or control sense-strand were generated from the following linearized plasmids: pBS-Xmab21 (antisense linearized NotI, trancribed from T7; sense linearized HindIII, transcribed from T3), Engrailed 2, Krox20, Emx2 (Maria Pannese), CS2+-Chordin (a gift of Stefano Piccolo), Xvent1, Xvent2 (a gift of Christof Niehrs). In single whole-mount hibridizations the probe was labeled with digoxigenin. Digoxigenin-labeled probes were immunolabeled with alkaline phosphatase-conjugated anti-digoxigenin antibody and with the BM Purple substrate (Boehringer). In the double whole-mount one probe was digoxigenin-labeled the second was labeled with fluorescein-UTP. Each probe was used at a concentration of 1 g/ml. Sequential detection with alkaline phosphatase-conjugated anti-fluorescein antibodies (1:8000) and anti-digoxigenin (1:2000) was done. The first color reaction was revealed with NBT/BCIP, while for the second color reaction the Vector Black kit II (Vector Laboratories) was used.

### Selection of peptide and production of Mab-21 antisera in rabbits

A 14aa C-terminal stretch from the mMab21l2 protein was chosen as immunogenic peptide to obtain antisera in rabbits. Peptide was synthesized, lyophilized and coupled to Keyhole limped hemocyanin (KLH). Coupled peptide was used for immunization of two rabbits. Immune and preimmune serum were controlled by ELISA using the same peptide coated at different serial dilutions. Immune serum was then purified by affinity chromatography: briefly, 20 mg peptide was coupled on CNBr-Sepharose-4B resin (Pharmacia), according to the standard procedure suggested. 50 ml antiserum diluted in 1:1 in 1X PBS was applied on the peptide/CNBr-Sepharose-4B over night at 4°C. After washing the resin with 50 ml PBS and 50 ml 3 M NaCl, bound antibodies were eluted using 100 mM Glycine pH 1.8. Amount, specificity and purity of the antiserum was tested by the Bradford assay, ELISA and SDS-PAGE/Coomassie staining. Purified antibody was tested for specificity in western blot on total protein extracts from cos-7 cells transfected with mMab21l2-myc fusion protein or mock vector, and on total brain extract from mouse embryos. One single band of 41 KDa was detected by anti Mab21l2 antibody. The same 41 kD band was detected by WB in lysates from transfected COS-7 cells, and was missing in untransfected cells. Cross-reactivity of anti mouse Mab21l2 antibody with the *Xenopus *Mab21l2 protein wastested by Western blot on total extracts from *X. laevis *embryos. The antibody did not work successfully in IHC or IP experiments, suggesting that it fails to recognize the native epitope.

### Western blotting

Protein concentrations in extracts were quantitated through Bradford assays. Extracts were gel-fractionated by denaturing polyacrylamide gel electropohoresis SDS-PAGE. Gels were transfered onto nitrocellulose filters. Even loading was confirmed by Ponceau staining of nitrocellulose filters. Imunostaining was conducted as described [39].

### Embryo injections

The vector *Xmab21-T7TS *was linearized with Eco*RI *and transcribed with the T7 RNA polimerase, N-MycMab21L2-CS2+ was linearized with Asp718 and transcribed with the SP6 RNA polymerase. The Flag-Smad1 was excised from the human construct Flag-Smad1-CMV5 (Courtesy of J. Massagué) and subcloned into the vector pCS2+. The Flag-Smad1-pCS2+ vector was linearized with Asp718 and transcribed with SP6. BMP4 expression constructs were a kind gift of N. Ueno. Capped mRNA was synthesized using the Ambion Message Machine kit according to manufacturer's instructions. Injections were performed in 4% Ficoll in 1X MMR. Embryos were injected animally at the one-cell or two-cell stage into one or both blastomeres. The amount of mRNA injected is given in the text.

### Luciferase activity assay

COS7 cells were maintained in DMEM Medium (Gibco Brl) supplemented with 10% FBS (Euro Clone), 2 mM L-glutamine (Euro Clone), 100 IU/ml penicillin, and 100 É g/ml streptomycin. Calcium phosphate transfection was performed in a 60 mm Petri dish with different amounts of the expression constructs, 5 É g of reporter plasmids, and 100 ng of pRL-TK (Promega) as an internal control. 18 h after transfection the cells were washed twice with PBS1X and the medium was replaced with DMEM Medium supplemented with 10% FBS, 2 mM L-glutamine (Euro Clone), 100 IU/ml penicillin, and 100 É g/ml streptomycin. Cells were harvested 36 h after the transfection, lysed, and assayed for luciferase activity (Dual Luciferase Reporter Assay System Kit, Promega). All luciferase assays were performed in triplicate.

### Immunoprecipitations

P19 cells were maintained in MEM alpha medium (Gibco Brl) supplemented with 10% FBS (Euro Clone), 2 mM L-glutamine (Euro Clone), 100 IU/ml penicillin, and 100 μg/ml streptomycin. Calcium phosphate transfection was performed in a 100 mm Petri dish with 10 μg of mycMab21l2 expression construct and 10 μg of flagSmad1 expression construct. 24 h after transfection the medium was replaced with MEM Alpha Medum supplemented with 1% FBS 2 mM L-glutamine (Euro Clone), 100 IU/ and 40 ng/ml BMP4 (R&D Systems). Cells were harvested 36 h after the transfection with TEN Solution (40 mM Tris pH7.5; 1 mM EDTA; 150 mM NaCl) and centrifugated for 15 minutes at 5000 RPM. Cell pellets were frozen at -80°C o/n, thawed at RT and resuspended in 5 volumes of Extraction Buffer (10 mM Hepes pH 7.9; 400 mM NaCl; 0,1 mM EGTA, 5% glicerol). The samples were centrifugated at 34000 RPM for 30 minutes at 4°C and the supernatants were harvested. The concentration of the proteins was determined by BCA assay (Pierce). *Xenopus laevis *lysates were obtained in the same way starting from injected embryos harvested by centrifugation.

After preclearing with 50 μl of protein G-sepharose o/n at 4°C, lysates were incubated with 25 μg of M2 anti-Flag monoclonal antibody (Sigma) and 50 μl of protein G-Sepharose o/n at 4°C. The resin was washed twice with Dilution Buffer (10 mM Tris-Cl pH8; 140 mM NaCl; 0.025% NaN_3_; 0.1% triton), once with TSA solution (10 mM Tris-Cl pH8; 140 mM NaCl; 0.025% NaN_3_) and once with 50 mM Tris-Cl pH 6.8. The protein complexes were eluted by addition of Sample buffer (Tris-Cl 125 mM pH 6.8; 0.1 M 2-mercaptoethanol; 2% SDS; 20% glycerol; 25 mg/ml Bromphenol Blue) followed by boiling for 5 minutes and separated on an SDS-polyacrylamide gel followed by anti-myc (1 μg/ml of clone n°9E10) immunoblotting. The secondary antibody was a goat anti-mouse antibody HRP-conjugated (1:30000 Bio-Rad). The blots were developed with the Supersignal West Pico reagent (Pierce).

### Purification of His-ZZ fusion proteins

The protocol was based on manufacturer's recommendations for Ni-NTA Agarose (Qiagen). His-ZZ fusion protein expression in bacterial cultures (37°C, BL21 *E. coli*) was induced by β-D-thiogalactopyranoside (IPTG) (0.4 mM for His-ZZ-Mab21; 1 mM for His-ZZ and for His-ZZ-Smad1) when the optical density (OD600) reached 0.7–0.9. Cells were harvested after 3 hours, resuspended in lysis buffer (50 mM NaH_2_PO_4_; 300 mM NaCl; lysozyme 2 mg/ml), and sonicated. The purification protocol was performed with Ni-NTA Agarose (Quiagen) beads and the elution with 250 mM Imidazole (Sigma).

### His-ZZ-Mab21l2 fusion protein pull-down assays

120 μg of His-ZZ-Mab21l2 fusion protein was incubated 1 h at 4°C with 25 μl of IgG-Sepharose beads (Amersham). The beads were recovered by centrifugation and washed ten times with 0.4 M KCl. The beads were incubated 1 h with 250 μg of precleared lysates of P19 treated and untreated with 40 ng/ml of BMP4. The beads were washed 5 times with 0.2 M KCl; the protein complexes were eluted by addition of Sample buffer followed by boiling for 5 minutes, and separated on an SDS-polyacrylamide gel for anti-Smad1 (1:2000 Santa Cruz) immunoblotting. The secondary antibody was a goat anti-rabbit HRP-conjugated antibody (Bio-Rad). The blots were developed with the Supersignal West Pico reagent (Pierce).

### His-ZZ-Smad1 fusion protein pull-down assays

120 μg of His-ZZ-Smad1 fusion protein was incubated 1 h at 4°C with 25 μl of IgG-Sepharose resin (Amersham). The beads were recovered by centrifugation and washed ten times with 0.4 M KCl.

The beads were incubated 1 h with 250 μg of precleared lysates of COS7 cells transfected with mycMab21l2. The beads were washed 5 times with 0.2 M KCl; the protein complexes were eluted by addition of Sample buffer,boiled for 5 minutes and separeted on a SDS-polyacrylamide gel for anti-myc immunoblotting.

### His-ZZ-Mab21l21 fusion protein pull-down assays with in-vitro translated proteins

120 μg of His-ZZ-Mab21l2 fusion protein was incubated 1 h at 4°C with 25 μl of IgG-Sepharose beads (Amersham) in Binding buffer (50 mM Tris pH8; 50 mM KCl; 5 mM MgCl_2_; 1 mM DTT; 0.2% NP-40, 10% glycerol). The beads were recovered by centrifugation and washed with Binding buffer. *In-vitro *translated, ^35^S-methionine-labeled Smad1 and Smad4 were prepared with the TNT coupled transcription/translation system (Promega). 45 μl of the TNT reaction were mixed with the His-ZZ-Mab21l2 bound to the IgG-Sepharose beads. The mixture was incubated for 2 hours at 4°C in Binding buffer. The Sepharose-protein complex was washed four times with Wash buffer (50 mM Tris pH7.5; 150 mM NaCl; 1 mM EDTA; 0.2% NP-40), eluted by addition of Sample buffer followed by boiling for 5 minutes and separated on SDS-polyacrylamide gels. Gels were fixed and stained with 50% methanol, 10% acetic acid and 0.1% Comassie Blue for 20 minutes. After the incubation with Sodium salicylate 1 M (Fluka) for 20 minutes to improve the radioactive signal the gels were dried and exposed to Kodak X-Omat film o/n at -80°C.

## Authors' contributions

DB did *in situ *hybridizations and *in vivo *overexpression experiments.

AB did pull-downs, immunoprecipitations and luciferase assays.

RL designed peptides and produced an anti MAB-21 polyclonal antibody.

VZ produced constructs for, and carried out DNA-binding assays and luciferase assays. In addition he helped design and evaluate all cellular assays.

GGC designed and coordinated the experimental work.

All authors read and approved the final manuscript
